# Targeting Epidermal Growth Factor Receptor by *MiRNA-145* Inhibits Cell Growth and Sensitizes NSCLC Cells to Erlotinib

**DOI:** 10.31557/APJCP.2019.20.9.2781

**Published:** 2019

**Authors:** Jamal Amri, Neda Molaee, Maryam Baazm, Hadi Karami

**Affiliations:** 1 *Molecular and Medicine Research Center,*; 2 *Traditional and Complementary Medicine Research Center, *; 3 *Department of Molecular Medicine and Biotechnology, *; 4 *Department of Anatomy, Faculty of Medicine, Arak University of Medical Sciences, Arak, Iran. *

**Keywords:** Apoptosis, growth, lung cancer, *MiRNA-145*, Tyrosine kinase inhibitor

## Abstract

**Background:**

Despite effective activity of epidermal growth factor receptor (EGFR) tyrosine kinase inhibitors (TKIs), such as erlotinib, all non-small cell lung cancer (NSCLC) patients eventually acquire resistance to these agents. Studies have demonstrated that down-regulation of *miRNA-145* leads to enhancement of* EGFR* expression, cell proliferation and metastasis. The aim of this study was to investigate the effect of *miRNA-145* on sensitivity of the A549 NSCLC cells to erlotinib.

**Methods:**

Quantitative real-time PCR was used to examine the effect of *miRNA-145* on *EGFR* expression. The effect of *miRNA-145* on cell growth and sensitivity the lung cancer cells to erlotinib was examined by trypan blue and MTT assays, respectively. The combination index was calculated using the non-constant method of Chou-Talalay. Apoptosis was determined by ELISA cell death assay.

**Results:**

We found that* miRNA-145 *was markedly suppressed the expression of EGFR and inhibited the cancer cell growth, relative to blank control and negative control miRNA (p<0.05). Pretreatment with *miRNA-145* synergistically enhanced the sensitivity of the lung cancer cells to erlotinib. Results of apoptosis assay revealed that *miRNA-145* can induce apoptosis and increase the erlotinib-mediated apoptosis.

**Conclusions:**

Our data demonstrate that *miRNA-145* play a critical role in the lung cancer cell growth, survival and EGFR-TKIs resistance possibly by regulation of *EGFR*. Therefore,* miRNA-145* replacement therapy can become a new therapeutic strategy in lung cancer.

## Introduction

lung cancer is one of the most common deadly diseases and leading cause of cancer-related deaths in the world (MacDonagh et al., 2015; Garinet et al., 2018). It can be divided into two different subtypes: small cell lung cancer (SCLC), which approximately account for 15% of lung cancer cases, and non-small cell lung cancer (NSCLC) which account for 85% (Wang et al., 2014; Zhang et al., 2014; Ashour Badawy et al., 2018). Despite significant advances in NSCLC therapies, the overall 5-year survival rate still remains at low level, due to the development of drug resistance (MacDonagh et al., 2015; Wang et al., 2015; Ma et al., 2016). The epidermal growth factor receptor (EGFR) also known as ErbB or HER1 is a member of the ErbB receptor tyrosine kinase (RTK) family that is overexpressed in many human malignancies including NSCLC (Normanno et al., 2006; Yoshida et al., 2010; Kumarakulasinghe et al., 2015). EGFR signaling triggers a network of signaling cascades, including the phosphoinositide (PI3K) pathway, Akt kinase pathway, STAT signaling pathway, Ras/Raf/MEK/ERK1/2 pathway, and the phospholipase C gamma protein pathway, which promotes tumor cell proliferation, invasion, survival, angiogenesis, metastasis and apoptosis resistance (Yoshida et al., 2010; Seshacharyulu et al., 2012; Kumarakulasinghe et al., 2015). Therefore, the *EGFR* has considered as a major therapeutic target in NSCLC.

Small-molecule* EGFR* tyrosine kinase inhibitors (TKIs) including gefitinib and erlotinib, showed effective activity in patients with NSCLC. Despite initial responses to EGFR-TKIs in NSCLC patients, the efficacy of these drugs is often limited by the development of drug resistance (Yoshida et al., 2010; Seshacharyulu et al., 2012; Antonicelli et al., 2013; Kumarakulasinghe et al., 2015). The poor clinical response of lung cancer cells to EGFR-TKIs is due to the inherent and acquired resistance of NSCLC cells to these agents, which is thought to occur via several mechanisms, including *PI3K *mutations, *T790M* mutation of EGFR, MET amplification, *HER-2* amplification, and transformation into a small-cell lung cancer phenotype (Antonicelli et al., 2013; Kumarakulasinghe et al., 2015). However, the other mechanisms of acquired resistance had remained unclear.

*MicroRNAs (miRNAs)* are a class of 21–25 nucleotides long, endogenous and non-coding RNAs that bind to 3’-untranslated region (3’-UTR) of mRNAs leading to transcript degradation or translational repression (Wang et al., 2014; MacDonagh et al., 2015; Abu-Duhier et al., 2018). *MiRNAs* have been shown to participate in a variety of cellular processes, including development, growth, proliferation, differentiation and apoptosis (Zhao et al., 2013; Ricciuti et al., 2014; Zhang et al., 2014). *MiRNA *deregulation and aberrant expression is a hallmark of many human cancers, including lung cancer, and they can be classified into oncogenic* miRNAs* (oncomirs) and tumor suppressive *miRNAs *(Markou et al., 2013; Zhang et al., 2014). For example, *miRNA-29* family expression is reduced in NSCLC cells, causing elevated expression of *DNMT3A* and *3B DNA* methyltransferases, increased tumorigenesis (Ricciuti et al., 2014; MacDonagh et al., 2015; Daei et al., 2018). In contrast, *miRNA-221* is overexpressed in lung cancer, leading to suppression of the *PTEN* and *PUMA*, enhanced invasiveness, migration, and drug resistance of the tumor cells (Wang et al., 2014; Zhang et al., 2014). Thus, *miRNAs *are emerging as potentially useful biomarkers in lung cancer research (Markou et al., 2013; Ricciuti et al., 2014; Zhang et al., 2014).

Studies have demonstrated that *miRNA-145* inhibits the expression of its targets, nucleoside X-type *motif-1 (NUDT-1)*, *c-Myc*, *eIF4e* and *CDK4*, supporting a tumor suppressor function of *miRNA-145* in lung cancer (Ricciuti et al., 2014; MacDonagh et al., 2015). Moreover, down-regulation of *miRNA-145 *in lung cancer tissue correlates with high levels of *EGFR*, reducing tumor growth, inducing cell cycle arrest in the G1/S phase and enhancing the cytotoxic effects of anti-tumor agents (Cho et al., 2011; MacDonagh et al., 2015; Wang et al., 2015). In the present study, we explored the effect of *miRNA-145 *on *EGFR* expression, cell growth and apoptosis in NSCLC cells. We hypothesized that* miRNA-145* would inhibit *EGFR* expression, and evaluated the synergy between *miRNA-145* and *EGFR-TKI* erlotinib in NSCLC cells.

## Materials and Methods

*Cell culture*

The human NSCLC cell line A549 (Pasteur Institute, Tehran, Iran) was cultured in RPMI-1640 (Sigma-Aldrich, St. Louis, MO, USA) supplemented with 10% fetal bovine serum (FBS) (Invitrogen, Carlsbad, CA, USA), 1% penicillin-streptomycin (Sigma-Aldrich), 2 mM of glutamine, and 1% sodium pyruvate at 37°C in a humidified atmosphere containing 5% CO2.

*Cell transfection*

The *miRNA-145* mimics and negative control (NC) miRNA were purchased from Dharmacon (Lafayette, CO, USA). Just before transfection, the A549 cells were cultivated in RPMI-1640 medium free of serum and antibiotics. *MiRNA* transfection (at a final concentration of 50 nM in all experiments) was done using Lipofectamine™2000 transfection reagent (Invitrogen, Carlsbad, CA, USA) according to the manufacturer’s protocol. In brief, miRNAs and lipofectamine (4 µl/ml of transfection medium) were diluted in Opti-MEM I medium (Invitrogen) separately and incubated for 5 min at room temperature. Then, the diluted miRNAs were combined with the diluted Lipofectamine and incubated for 20 min at room temperature. Subsequently, the complexes were then added to the culture medium. The cells were then incubated for 6 h at 37^o^C in a humidified CO_2_ incubator. Next, complete growth medium containing FBS (final FBS concentration of 10%) was added and cells were cultured under the above mentioned conditions. After 24 and 48 h, suppression of *EGFR* gene expression was measured by real-time quantitative PCR (qRT-PCR).

*QRT-PCR*

Total RNA was extracted from cultured cells by RNA extraction kit (Takara Bio Inc., Kusatsu, Shiga, Japan) according to the manufacturer’s recommendations. Complementary DNA (cDNA) was prepared from 0.5 µg of total RNA using PrimeScript Reverse Transcriptase and oligo-dT primer (Takara Bio Inc.) according to manufacturer’s instructions. Quantitative real-time PCR was performed with the LightCycler 96 System (Roche Diagnostics GmbH, Mannhein, Germany) using the SYBR Green qPCR MasterMix (Yekta Tajhiz Azma, Tehran, Iran). The reaction system of real-time PCR was: 1 µl of cDNA template, 0.2 µM of primers, 10 µl of SYBR green reagent and 7 µl of nuclease-free distilled water. The sequences of PCR primers were as follows: forward, 5’- CTACAATGAGCTGCGTGTG-3’, reveres, 5’- GTCTCAAACATGATCTGGGTC-3’, for β-actin, and forward, 5’-TTTACAGGAAATCCTGCATGG-3’, and reverse, 5’- TCACTGCTGACTATGTCCC-3’, for EGFR. The PCR conditions were as follows: initial denaturation at 95°C for 10 min followed by 40 cycles of denaturation at 95°C for 10 sec, annealing at 57°C for 20 sec and 72 ^o^C for 20 sec. The relative EGFR expression level was calculated according to the 2 ^-(∆∆Ct)^ method (Livak and Schmittgen, 2001), using β-actin as the reference gene.

*MTT assay*

The effect of *miRNA-145* on the sensitivity of lung cancer cells to erlotinib (Sigma- Aldrich) was determined using 3-(4, 5-Dimethylthiazol-2-yl)-2, 5 Diphenyltetrazolium Bromide (MTT) assay. The experiment was subdivided into eight groups: *miRNA-145* mimics, NC miRNA, erlotinib, miRNA-145 mimics and erlotinib, NC miRNA and erlotinib, miRNA blank control, erlotinib blank control and combination blank control. In brief, cells were cultured at a density of 5×10^3^ cells/well in 96-well cell culture plates and then transfected with miRNAs. After 6 h of incubation, the cells were treated with different concentrations of erlotinib (0, 2, 4, 8, 16, 32 and 64 µM). After 24 and 48 h of transfection, the cytotoxicities of the treatments were determined using the MTT cell assay kit (Roche Diagnostics GmbH, Mannheim, Germany) according to the manufacturer’s instructions. The amount of formazan dye in each well was measured by quantifying its absorbance (A) at 570 nm (with a reference wavelength of 650 nm) using a microplate spectrophotometer (Awareness Technology, Palm City, FL, USA). The survival rate (SR) was calculated from the following equation: SR (%) = (A Treatment /A Control) ×100%. Half maximal inhibitory concentration (IC_50_) values were calculated using GraphPad Prism 6.01 software (GraphPad Software Inc., San Diego, CA, USA).

*Combination effect analysis*

The combination index (CI) theorem of Chou-Talalay was used to determine the interaction between erlotinib and *miRNA-145* (Chou and Talalay, 1984). The results obtained from the MTT assay were converted to Fraction affected (Fa; range 0-1; where Fa = 0 is 100% cell survival and Fa = 1 is 0% cell survival) and analyzed using CompuSyn program from Combosyn (Paramus, NJ, USA). Synergistic, additive, and antagonistic effects are defined by CI<1, CI=1 and CI>1, respectively.

*Cell growth assay*

For the assessment of cell growth, lung cancer cells (6×10^4^ cell/well) were treated with *miRNA-145* in 24-well cell culture plates and then incubated for 24-120 h. After indicated time points, cells were harvested and stained with 0.4% trypan blue solution (Merck KGaA, Darmstadt, Germany) for 5 min. Following on, the number of viable cells (N, unstained cells) was quantified every day under an inverted microscope (Nikon Instrument Inc., Melville, NY, USA) using a hemocytometer. The percentage of viable cells was calculated according to the following formula: Cell viability (%) = (N Test /N Control) ×100. 

*Determination of apoptosis*

Apoptosis was assessed with a Cell Death Detection ELISA PLUS kit (Roche Diagnostics GmbH) that quantifies mono- and oligonucleosomes in the lysates of apoptotic cells. The A549 lung cancer cells were cultured at a density of 1×10^5^ cells/well in 12-well cell culture plates and treated with erlotinib (IC_50_ doses of 24 and 48 h), *miRNA-145* or *NC miRNA*, and their combination, as described in the MTT assay section. In brief, the cells were lysed in lysis buffer and centrifuged for 10 min at 200 g. Then, 80 µl of immunoreagent containing anti-DNA-peroxidase and anti-histone-biotin and 20 µl of the cell supernatant were added to each well of streptavidin-coated plate. After 2 h of incubation in room temperature, 100 µl of ABTS solution was added to each well an incubated on a plate shaker at 250 rpm for 15 min. After color development, 100 µl of ABTS stop solution were added to each well and the absorbance at 405 nm was measured with the microplate reader. The apoptosis rate was expressed as the fold increase in apoptosis relative to the control group, which was considered as 1.

*Statistical analysis*

All data were presented as mean± standard deviation (SD) of three independent experiments. Statistical analysis was performed by ANOVA followed by Bonferroni’s test. Values of p less than or equal to 0.05 was considered significant. All data analyses were performed using GraphPad Prism software.

## Results

*MiRNA-145 down-regulated EGFR mRNA levels in lung cancer cells*

**Figure 1:**
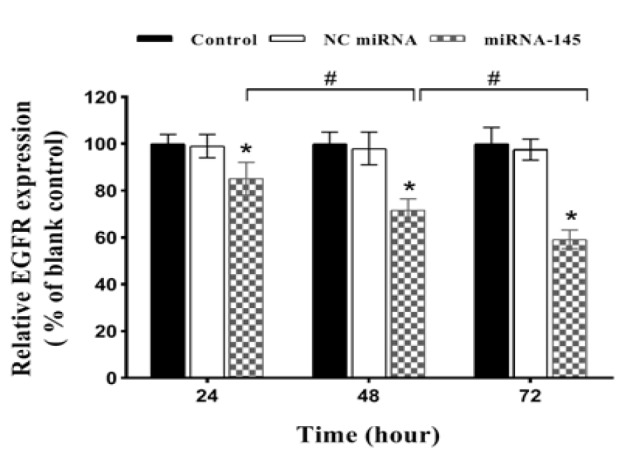
Expression of *EGFR* in A549 Lung Cancer Cells Transfected with *miRNA-145*. To measure the expression of *EGFR* in lung cancer cells, the cells were transfected with *miRNA-145* and negative control (NC) miRNA for 24, 48 and 72 h. Then, the *EGFR* gene expression was measured by quantitative real-time PCR and 2 ^- (∆∆Ct)^ method. Data are expressed as mean±SD of three independent experiments; *p<0.05 significantly different from corresponding blank control and *NC miRNA*. *#p<0.05*

First, we examined the effect of *miRNA-145* on EGFR gene expression in A549 cells by qRT-PCR. Relative *mRNA* expression was calculated in relation to the blank control, which was set at 100%. As shown in Figure 1, transfection of tumor cells with *miRNA-145* led to a marked time-dependent reduction of* EGFR mRNA *level (p<0.05; relative to the blank control). At 24, 48 and 72h after the transfection, the relative expression of *EGFR mRNA* levels were 85.11%, 71.57% and 59.23%, respectively (p<0.05). Notably, there was no difference in *EGFR mRNA* levels among NC miRNA and blank control group (p>0.05; Figure 1).

**Table 1:** Half Maximal Inhibitory Concentration (IC_50_) of Erlotinib Alone and in Combination with miRNAs in A549 Cells

Treatment	IC_50_	
	24 h	48 h
Erlotinib	21.47 ± 1.18	14.26 ± 1.83
NC miRNA and erlotinib	20.35 ± 1.11#	13.17 ± 1.42#
miRNA-145 and erlotinib	11.36 ± 2.13*	7.63 ± 0.66*

IC_50_ values were calculated by sigmoidal dose-response (variable slope) model using GraphPad Prism software. Results expressed as the mean±SD (n=3). *p<0.05 versus erlotinib; #p>0.05 relative to the erlotinib.

**Figure 2:**
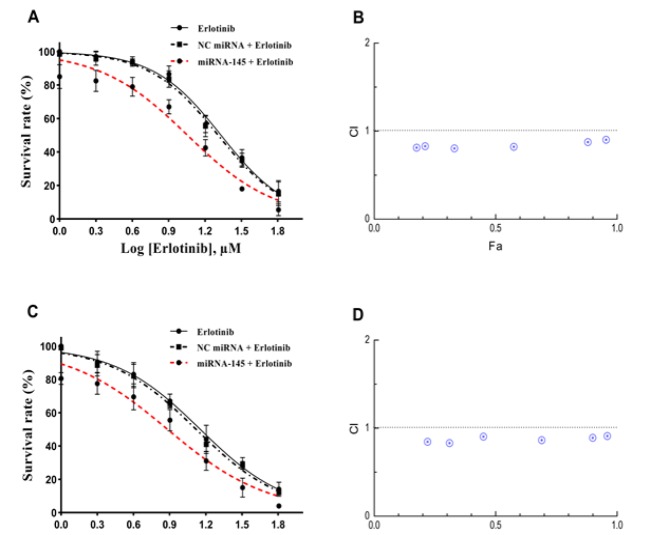
Effect of miRNA-145 on Sensitivity of the Lung Cancer Cells to Erlotinib. Cells were treated with *miRNA-145 *(50 nM) and various concentrations of erlotinib for 24 h (A and B) and 48 h (C and D). After treatment, cell survival was determined by the MTT assay as described in the method section. Cell survival curves were plotted using Prism 6.01 software. The data represent mean±SD (n=3). Data from three independent experiments were used to plot the combination index (CI) versus fractional effect (Fa) according the method of Chou and Talalay. Dashed lines indicate CI=1.

**Figure 3:**
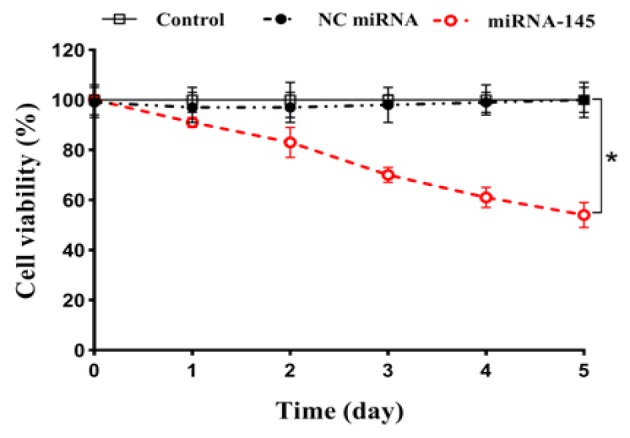
Growth Curve of A549 Cells Transfected with miRNA-145 and Negative Control (NC) miRNA. Cell growth was measured using trypan blue exclusion assay over a period of 5 days. The results are expressed as mean±SD (n=3). *p<0.05 versus blank control or NC miRNA

**Figure 4:**
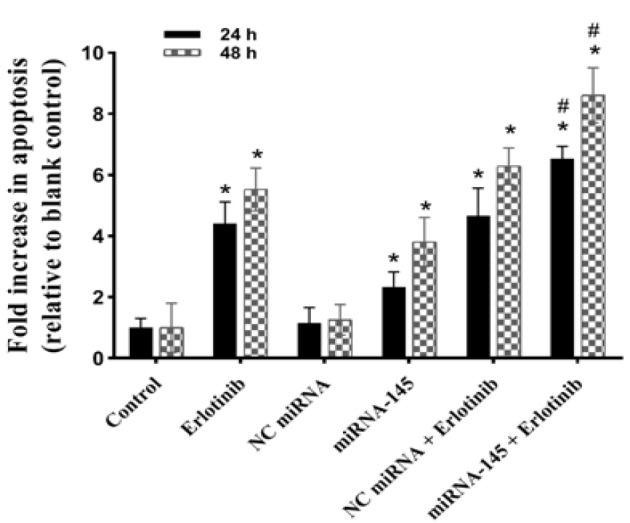
Combination Effects of miRNA-145 and Erlotinib on Lung Cancer Cell Apoptosis. Cells were transfected with miRNA-145 (50 nM) and negative control (NC) miRNA (50 nM) for 6 h. Then, erlotinib (IC_50_ doses of 24 and 48 h) was added to the cells. After 24 and 48 h of transfection, the apoptosis was determined using ELISA cell death assay. The data presented are mean±SD (n=3) of independent experiments; *p<0.05 compared with blank control or NC miRNA; *#p<0.05 *versus miRNA-145 or erlotinib

*MiRNA-145 increased sensitivity to erlotinib in A549 lung cancer cells*

To assess whether suppression of *EGFR* expression by *miRNA-145* could enhance the sensitivity of lung cancer cells to erlotinib, a combination treatment with *miRNA-145* and erlotinib was performed. The results showed that single treatment with erlotinib induced cytotoxicity in a dose-dependent way. At 24 and 48 h after transfection, *miRNA-145* alone significantly decreased the cell survival rate to 85.07% and 80.56% respectively, compared with the blank control (Figure 2A and 2C; p<0.05). Furthermore, combination therapy further decreased the survival rate of the A549 cells compared with *miRNA-145* or erlotinib monotreatment (p<0.05). In the presence of *miRNA-145*, the IC_50_ values of erlotinib drastically lowered from 21.47 µM to 11.36 µM and 14.26 µM to 7.63 µM respectively, after 24 and 48 h (Table 1). Notably, NC miRNA had a minimal effect on the chemosensitivity of the cells compared with miRNA-145 (p>0.05; Figure 2 and Table 1).

*The combination effect of miRNA-145 and erlotinib on A549 cells was synergistic*

To further explore whether the combination of miRNA-145 and erlotinib is synergistic, we performed the combination index analysis using the non-constant method of Chou-Talalay. Our results showed that the combination effects of *miRNA-145* (50 nM) and erlotinib (2-64 µM) were synergistic in A549 cells with the CI values of less than 1 in all concentrations of erlotinib (Figure 2B and 2D). CI–Fa plots indicated that the strongest synergistic effects of 24 h (CI=0.80) and 48 h (CI=0.83) of treatment were observed at 8 and 4 µM of erlotinib with Fa levels of 0.33 and 0.31, respectively.

*Up-regulation of miRNA-145 inhibited cell growth*

As down-regulation of *miRNA-145* is correlated with survival of lung cancer; we therefore sought to test whether up-regulation of* miRNA-145* could suppress the growth of lung cancer cells. The *A549* cells were transfected with *miRNA-145* and *NC miRNA* and cell viability was determined by trypan blue assay. Our results showed that, compared with the blank control, *miRNA-145 *dramatically inhibited the growth of cancer cells over a period of 5 days (p<0.05; Figure 3). Twenty-four hours after transfection, the cell viability decreased to 91.39 % and then to a further 54.44% at the end of the experiment (day 5). However, no significant differences in cell growth was observed between the *NC miRNA* transfected cells and the blank control group (p>0.05; Figure 3).

*Up-regulation of miRNA-145 augmented erlotinib-induced apoptotic cell death*

To investigate whether the observed chemosensitizing effects of the *miRNA-145* were linked to a higher number of apoptotic cells, the effects of *miRNA-145 *and erlotinib alone and in combination on apoptosis, were examined using an ELISA cell death assay. Figure 4, demonstrates that 24 h exposure of *A549* lung cancer cells with miRNA-145 or erlotinib enhances apoptosis by 2.33 fold and 4.42 fold, respectively, compared to the blank control (p<0.05). In addition, combination of both agents further promotes the degree of apoptosis to 6.54 fold (p<0.05, compared with either erlotinib alone or *miRNA-145 *alone). On the other hand, 48 h treatment of *A549* cells with *miRNA-145* or erlotinib alone, enhanced apoptosis by 3.81 and 5.53 fold, respectively, relative to the blank control group (p<0.05). Also, the combination of *miRNA-145* and erlotinib further enhanced the induction of apoptosis to 8.61 fold during same period of time (p<0.05, relative to the blank control or monotherapy). However, treatment with *NC miRNA* alone or in combination with erlotinib showed no distinct changes in the extents of apoptosis compared with the* miRNA-145* or etoposide treated cells, respectively (Figure 4; p>0.05). These results indicate that *miRNA-145* sensitizes the lung cancer cells to erlotinib partially via enhancement of apoptosis. 

## Discussion

Despite the substantial progress in the treatment of lung cancer, 5-year survival rates remain at low level, largely owing to the development of drug resistance (MacDonagh et al., 2015; Wang et al., 2015). Therefore, development of new effective treatment for improved therapy and survival is necessary. Studies have demonstrated that overexpression of EGFR is enhanced cell survival, proliferation, metastasis and angiogenesis in a variety of human malignancies including NSCLC (Yoshida et al., 2010; Seshacharyulu et al., 2012; Kumarakulasinghe et al., 2015). The poor clinical response of lung cancer cells to anti-EGFR therapies is due to the primary and secondary resistance of NSCLC cells to these drugs (Antonicelli et al., 2013; Kumarakulasinghe et al., 2015). However, the exact mechanisms of resistance had remained unclear. In the present study, we examined the effect of *miRNA-145 *on* EGFR *expression, cell growth and sensitivity of lung cancer cells to erlotinib. 

The results of RT-qPCR showed that treatment with miRNA-145 drastically lowered the *EGFR mRNA *expression levels over a 3-day period. These findings suggest that *miRNA-145* could effectively block the expression of the *EGFR*, partially via cleavage of the corresponding mRNA. The cell viability assay revealed that the up-regulation of *miRNA-145* significantly decreased the viability of tumor cells compared with the blank control during a 5-day period. These findings show that *miRNA-145* may play a critical role in the growth and survival of lung cancer cells through inhibition of *EGFR*. The MTT assay demonstrated that pretreatment with* miRNA-145* markedly augmented the cytotoxicity of erlotinib in *A549* cells, and subsequently the IC_50_ value of erlotinib was significantly decreased. Combination study results showed a clear synergistic interaction between miRNA-145 and erlotinib at all concentrations of erlotinib.

To further explore the role of *miRNA-145* in the drug resistance of lung tumor cells, we tested the impact of* miRNA-145* on the apoptotic effect of erlotinib. Apoptosis assay findings showed that monotherapy with erlotinib resulted in significant apoptosis in *A549 *cells. Moreover, miRNA-mediated inhibition of EGFR triggered remarkable spontaneous apoptosis and increased sensitivity of the cancer cells to erlotinib-mediated apoptosis. In contrast, treatment with negative control miRNA or lipofectamine did not change the *EGFR *expression, cell proliferation and chemosensitivity of the cells, illustrating the specific impact of *miRNA-145*. These results proposed that down-regulation of EGFR by miRNA-145 could sensitize the lung cancer cells to erlotinib.

While accumulating evidence points to the direct involvement of miRNAs in regulation of tumor pathogenesis and resistance, such findings represent the potential of manipulating *miRNAs* to sensitize tumor cells to the effects of radiation, chemotherapy and targeted therapies (MacDonagh et al., 2015). *MiRNA-145* is a tumor suppressor that is transcripted from a bicistronic gene cluster on chromosome 5. Previous studies reported that *miRNA-145* was strongly down-regulated in different types of cancers such as gastric, colon, bladder, breast, cervical, leukemia, prostate and lung (Cho et al., 2009; Duan et al., 2014; Ricciuti et al., 2014). Other studies showed that the down-regulation of *miRNA-145* in human lung adenocarcinoma was associated with *EGFR* amplification and its transfection inhibited cancer cell growth through the suppression of its targets, *EGFR* and NUDT-1 (Cho et al., 2011; Seshacharyulu et al., 2012). Chen and colleagues (2010) also found that transfection of the *miRNA-145 *to *A549 *and H23 NSCLC cell lines suppressed the expression of *CDK4*, *eIF4e* and *c-Myc *genes, induced cell cycle arrest and reduced growth and cisplatin resistance. Consistent with these reports, our data confirmed that transfection of *miRNA-145* significantly inhibited *EGFR *expression and enhanced the cytotoxic effects of erlotinib in *A549* cell line.

The *EGFR* expression has been reported to increase in many cancers (Yoshida et al., 2010; Kumarakulasinghe et al., 2015). Two previous studies demonstrated that *miRNA-7* and *miRNA-146a* can suppress the expression of* EGFR* gene and increase the sensitivity of the lung cancer cells to E*GFR-TKIs *(Rai et al., 2011; Chen et al., 2013). Other studies showed that *miRNA-145* expression reduced in lung cancer cells, causing elevated *c-MYC*, *NUDT1*, *OCT4* and *EGFR* expression, increased tumor cell proliferation, metastasis and migration (Liu et al., 2009; Chen et al., 2010; Cho et al., 2011; Yin et al., 2011; Guan et al., 2012). In our study, we found that *miRNA-145 *can inhibit the cell growth and enhance the sensitivity of the NSCLC cells to erlotinib by targeting *EGFR*. 

Together, we have demonstrated that *miRNA-145 *plays a critical role in the growth, apoptosis and drug-resistance of lung cancer cells. Knockdown of *EGFR* by *miRNA-145 *induced apoptosis and sensitized the A549 lung tumor cells to erlotinib in a synergistic way. Our data suggest that therapeutic delivery of *miRNA-145* may be considered as a new remedial approach to overcome the *EGFR-TKIs *resistance in lung cancer cells.
